# Assessing Boron-Pleuromutilin AN11251 for the Development of Antibacterial Agents

**DOI:** 10.3390/molecules28124628

**Published:** 2023-06-08

**Authors:** Ming-Jie Han, Miaomiao Pan, Genhui Xiao, Ying Yuan, Shawn Chen, Zhiyang Zou

**Affiliations:** 1 Department of DMPK & Tox, Global Health Drug Discovery Institute, Zhongguancun Dongsheng International Science Park, Beijing 100192, China; 2 Department of TB Biology, Global Health Drug Discovery Institute, Zhongguancun Dongsheng International Science Park, Beijing 100192, China

**Keywords:** pleuromutilins, AN11251, antibacterial, anti-*Wolbachia*, in vitro ADME, pharmacokinetics, dose prediction

## Abstract

Pleuromutilins are a group of antibiotics derived from the naturally occurring compound. The recent approval of lefamulin for both intravenous and oral doses in humans to treat community-acquired bacterial pneumonia has prompted investigations in modifying the structure to broaden the antibacterial spectrum, enhance the activity, and improve the pharmacokinetic properties. AN11251 is a C(14)-functionalized pleuromutilin with a boron-containing heterocycle substructure. It was demonstrated to be an anti-*Wolbachia* agent with therapeutic potential for Onchocerciasis and lymphatic filariasis. Here, the in vitro and in vivo PK parameters of AN11251 were measured including PPB, intrinsic clearance, half-life, systemic clearance, and volume of distribution. The results indicate that the benzoxaborole-modified pleuromutilin possesses good ADME and PK properties. AN11251 has potent activities against the Gram-positive bacterial pathogens tested, including various drug-resistant strains, and against the slow-growing mycobacterial species. Finally, we employed PK/PD modeling to predict the human dose for treatment of disease caused by *Wolbachia*, Gram-positive bacteria, or *Mycobacterium tuberculosis*, which might facilitate the further development of AN11251.

## 1. Introduction

The emergence and spread of antimicrobial resistance have become a global threat to public health, increasingly reducing the options available to treat life-threatening bacterial infections. Modern medical procedures such as cardiovascular surgery, joint replacement, tooth extraction, and organ transplantation cannot be safely and successfully operated without effective antibacterial agents. However, nosocomial infections caused by vancomycin-resistant enterococci (VRE) are rapidly rising worldwide. Drug-resistant pathogens such as methicillin-resistant *Staphylococcus aureus* (MRSA) are routinely found outside the clinic. Therefore, there is an urgent need for the development of new antibacterial treatments with high efficacy and low resistance rates.

Pleuromutilins are semi-synthetic antibiotics derived from natural tricyclic diterpenoid pleuromutilin produced by an edible mushroom, *Pleurotus mutilus* [1,2]. Pleuromutilins inhibit bacterial protein synthesis by binding to the 50S ribosomal subunit at the peptidyl transferase center [3,4,5]. Pathogens that are resistant to other major antibiotic classes do not have cross-resistance to pleuromutilins [6]. Early pleuromutilin derivatives, such as tiamulin [7,8] and valnemulin [8], were developed as veterinary medicine to treat respiratory and intestinal infections in farm animals. Retapamulin was the first pleuromutilin for humans, used topically to treat impetigo and skin infections caused by *Staphylococcus aureus* and *Streptococcus pyogenes* [9,10]. Recently, lefamulin was approved by the US Food and Drug Administration (FDA) and the European Commission for the treatment of community-acquired bacterial pneumonia [10,11]. Lefamulin marketed in oral and intravenous formulations demonstrated clinical efficacy and an acceptable safety profile, suggesting the general tolerability and low risk of side effects of pleuromutilins in other potential therapeutic areas. The pleuromutilin derivatives often bear modifications at the C14 side chain such as the incorporation of a quaternary amine or amino acid.

AN11251 is a benzoxaborole-modified pleuromutilin for improving the pharmacologic, physicochemical, and pharmacokinetic properties of this drug [12,13]. AN11251 is a pre-clinical candidate for treatments of Onchocerciasis or river blindness and lymphatic filariasis. The diseases are caused by parasitic nematodes which carry obligate endosymbiotic *Wolbachia* bacteria. AN11251 can kill *Wolbachia*-infected cells and adult worms in vitro, and the in vivo efficacy was established with the infection mouse model to be superior or comparable to existing antibacterial treatments. Its great efficacy and lessened risk of drug resistance warrant further consideration for therapeutics against bacterial pathogens of global and public health concerns.

We set out to investigate the PK properties and antibacterial potency of AN11251 for the development of an antibacterial agent with new indication. Here in this paper, the ADME (absorption, distribution, metabolism, and excretion) and PK (pharmacokinetics) profile were first measured, such as the PPB (plasma protein binding), metabolic stability, half-life, volume of distribution [14], and so on. We also demonstrated the good to excellent activities of AN11251 against Gram-positive bacteria and *Mycobacterium*. Finally, the human dose of AN11251 for antibacterial use was predicted using a PK/PD (pharmacokinetics and pharmacodynamics) model [15].

## 2. Results and Discussion

### 2.1. Plasma Protein Binding

Plasma protein binding (PPB) related to drug lipophilicity plays an important role in drug efficacy and toxicity [16]. In general, the more lipophilic a drug is, the more significant the PPB becomes. The incorporation of boron at the C14 site of pleuromutilin could improve its oral bioavailability. AN11251 presents good permeability (apparent permeability (Papp) = 14.1 × 10^−6^ cm/s) at the MDR1-MDCK assay [12]. AN11251 exhibits high PPB values in different animal species because of the high logP (4.5). The bound fractions in mouse and human are 96.6% and 97.6%, respectively. The PPB value of rats is so high that we did not detect the unbound fraction. That PPB values are different between species is common. All the results reflect AN11251′s lipophilic nature.

### 2.2. In vitro Metabolic Stability

**Figure 1:**
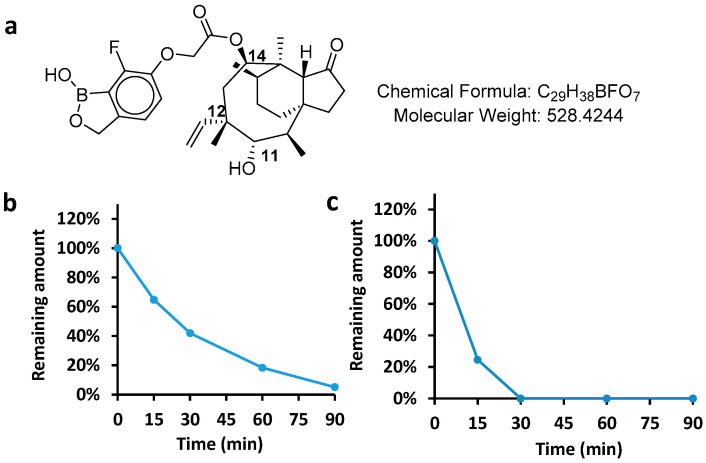
The chemical structure of AN11251 (**a**) and in vitro metabolic stability of AN11251 in (**b**) human hepatocytes and (**c**) rat hepatocytes.

Metabolic stability is defined as the susceptibility of a drug to biotransformation, and expressed as intrinsic clearance or terminal half-life [17,18]. The metabolic stability of AN11251 was determined in both liver microsomes and hepatocytes. The results presented different intrinsic clearances in different animal species. The clearance of AN11251 was 299.9 mL/min/kg in human liver microsomes, and 180.2 mL/min/kg in human hepatocytes, which indicated that the hepatocyte clearance was lower than the liver microsomal clearance. As liver microsomal stability assays only contain the NADPH cofactor with no other cofactors, when hepatocyte intrinsic clearance is higher than liver microsome, the non-CYP metabolic enzyme may be involved in the biotransformation, such as UGTs, SUITs, AO, and ADH/ALDH. On the other hand, when the liver microsomal clearance is higher than the hepatic clearance, membrane permeability may limit the apparent metabolic rate. This permeability limitation could be caused by efflux transporters on the human hepatocyte membrane. However, the hepatocyte membrane permeability is different between in vitro and in vivo experiments, which would not affect in vivo compound dispositions towards the liver [19]. In rat studies, the situation could be different. The intrinsic clearance of AN11251 at 876.5 mL/min/kg in rat hepatocytes was higher than 332.6 mL/min/kg of rat liver microsomes, indicating that there might be a phase II metabolism in hepatocytes. The elimination process is shown in Figure 1.

### 2.3. Pharmacokinetics of AN11251 in Rats

**Figure 2:**
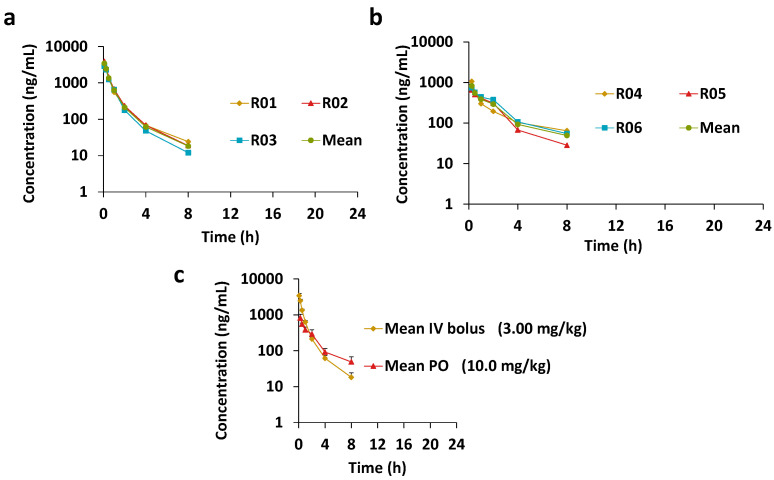
The plasma concentration–time curve of intravenous (**a**) and oral (**b**) administration. (**c**) The mean plasma concentration–time profile of intravenous (IV) and oral administrations (PO, per os).

Although the high plasma protein binding helps to reduce the compound from metabolism, the high metabolism leads pleuromutilins to a poor PK. There is plenty of room to promote the metabolism stability, bioavailability, etc. [20,21]. Great efforts on pleuromutilin chemical modification have been carried out, resulting in over a hundred compounds in this serial [22], but most of them might have poor PK profiles. The pharmacokinetic properties of AN11251 were investigated in rats following intravenous (IV) administration at a dose of 3 mg/kg and oral (PO) administration at a dose of 10 mg/kg (Figure 2). Surprisingly, the PK properties of AN11251 in rats were reviewed as moderate to good (Table 1), which would be one of only a few cases. As shown in the plasma concentration–time curve, the drug candidate AN11251 was eliminated within 8 h, and generated a mean half-life T1/2 of 1.75 h, and mean residence time (MRT) of 1.22 h. The system clearance was modest and exhibited 19.8 mL/min/kg, 36% of the liver blood flow, which was consistent with the intrinsic clearance in microsomes. The Vdss was 1.44 L/kg, indicating that AN11251 had good lipophilicity and permeability to be distributed to the body fluid and tissues. After PO administration, AN11251 was absorbed rapidly and reached the maximum drug concentration at 0.25 h, owing to its good permeability and solution formulation. The oral bioavailability was 19.2% as a high intrinsic clearance (332.6 mL/min/kg) may generate a strong first-pass metabolism. The PK properties of AN11251 were different between animal species tested. In the previous report, the mouse assay with the same formulation exhibited a very good PK profile, a lower systemic clearance (8.4 mL/min/kg), a larger volume of distribution Vdss (4.186 L/kg), and a higher bioavailability (61%) [12]. When thiazole-pyridine was incorporated into the C14 of pleuromutilin, the PK property was unfavorable, with systemic clearance 31.6 mL/min/kg [3]. In addition, the thioether pleuromutilin derivatives also suffered from low exposure and high clearance [4]. Therefore, the boron-heterocycle-modified pleuromutilin AN11251 showed reasonable to attractive PK properties.

### 2.4. Antibacterial Activities of AN11251

The MIC determination of AN11251 against Gram-positive, Gram-negative bacterial strains, and mycobacterial strains shows the compound has very good inhibitory activity against Gram-positive bacteria and fastidious microorganisms such as *H. influenzae* (Table 2) as reported for other pleuromutilins. It has no inhibitory activity in vitro against many Gram-negative bacteria. AN11251 is a potent growth inhibitor of the slow-growing mycobacteria, *M. bovis* BCG and *M. tuberculosis* H37Rv, but has little inhibitory activity against the fast-growing mycobacteria such as *M. smegmatis* and *M. abscessus*. AN11251 has a level of cytotoxicity, shown by the concentration that reduced the viability of a Vero6 cell line (from African green monkey kidney) by 50% at 27 µg/mL, which gives the therapeutic index (CC50/MIC) of AN11251 in the range of ~700:1 to ~30:1, indicating the margin of safety is large to reasonable. The SAR investigation has demonstrated that optimal activity was generated by linking the benzoxaborole to the pleuromutilin core [12]. AN11251 could efficiently treat Gram-positive bacteria, *Wolbachia*, and tuberculosis.

### 2.5. Prediction of Human Dose Using PK/PD Model

**Figure 3:**
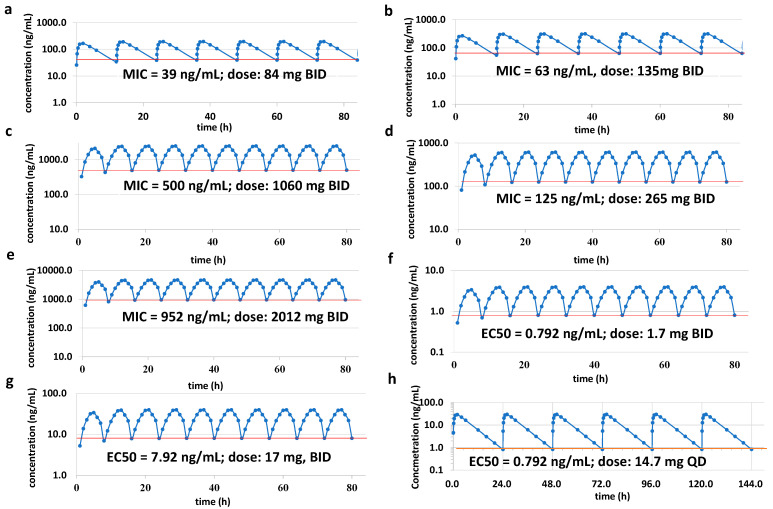
Prediction of human dose of AN11251 using PK/PD modeling for Gram-positive bacteria such as (**a**) *Staphylococcus aureus* ATCC 29213 and *Staphylococcus aureus* ATCC 700698; (**b**) *Staphylococcus aureus* SAU-0167, *Staphylococcus aureus* SAU-9922, *Staphylococcus epidermidis* SEP-1024, and *Streptococcus pyogenes* SPY-0253; (**c**) *Streptococcus pneumoniae* SPN-1169; (**d**) *Enterococcus faecium* EFA-0221; (**e**) *Mycobacterium tuberculosis H37Rv* ATCC 27294; (**f**) *Wolbachia*-infected LDW1 cells (wMel); (**g**) *Wolbachia*-infected C6/36 cells (wAlb); and (**h**) *Wolbachia*-infected LDW1 cells (wMel) for QD dosing.

The human dose of AN11251 was predicted using a one-compartment pharmacokinetic/pharmacodynamic (PK/PD) model. The PK/PD modeling links the concentration–time profile assessed by pharmacokinetics to the response intensity quantified by pharmacodynamics. The PK/PD modeling could thus elucidate the causative relationship between drug concentration and efficacy and provide a better understanding of the mechanism that results in the drug’s effect. This model integrates drug-specific parameters, such as systemic clearance, the volume of distribution, and bioavailability, with a drug-independent structural model consisting of anatomical compartments. The human systemic clearance was estimated through the simple allometric scaling using rat PK systemic clearance. The systemic clearance of humans was predicted as 2.833 mL/min/kg, 11.9 L/h for the 70 kg human body weight. The human volume of distribution was 1.029 L/kg, also estimated through simple allometric scaling using rat volume of distribution data. In the PK/PD modeling, the absorption rate constant ka was defined as 1.5, the bioavailability F was 0.19, and the half-life was 4.2 h. The PK/PD model was based on the predicted human PK profile; a dosing regimen was defined to keep drug concentrations above the threshold of efficacy (e.g., MIC) (Figure 3). AN11251 had a very good anti-*Wolbachia* activity. According to the EC50 measured on infected cells, the human dose was predicted to be only 1.7 mg (BID) for infected LDW1 cells and 17 mg (BID) for C6/36 cells. If humans were administrated once a day, the predicted dose could be as low as 14.7 mg once a day. For the Gram-positive bacteria *Staphylococcus aureus* ATCC 29213 and *Staphylococcus aureus* ATCC 700698, the predicted daily dose of AN11251 was lower than 84 mg (1.2 mg/kg) twice a day according to the MIC value lower than 0.039 µg/mL. For the bacteria *Staphylococcus aureus* SAU-0167, *Staphylococcus aureus* SAU-9922, *Staphylococcus epidermidis* SEP-1024, and *Streptococcus pyogenes* SPY-0253 with the MIC 0.063 µg/mL, the predicted human dose was 135 mg (1.9 mg/kg) twice a day. For the bacterium *Streptococcus pneumoniae* SPN-1169 with the MIC 0.5 µg/mL, the predicted human dose was 1060 mg/kg (15.1 mg/kg) twice a day. For the bacterium *Enterococcus faecium* EFA-0221 with the MIC 0.125 µg/mL, the predicted human dose was 265 mg/kg (3.8 mg/kg) twice a day. For the *Mycobacterium tuberculosis H37Rv* ATCC 27294 with the MIC 0.925 µg/mL, the predicted human dose was 2012 mg (28.7 mg/kg) twice a day. The predicted results demonstrated that AN11251 was effective in reducing the Gram-positive bacteria and the *Mycobacterium tuberculosis* H37Rv. For the incorporation of boron-heterocycle into pleuromutilin, AN11251 presented good exposure in plasma after PO administration with beneficial ADME and pharmacokinetic properties. Meanwhile, the improved antibacterial activity of AN11251 makes the PK/PD prediction favorable.

## 3. Materials and Methods

### 3.1. Materials

AN11251 was provided by Calibr at Scripps Research (San Diego, CA, USA). All the other chemicals were purchased from Sigma (St Louis, MO, USA) without further purification. Rats were purchased from Charles River (Wilmington, NC, USA) and fasted overnight before dosing. Yeast Extract (OXOID, from Thermo Fisher (Waltham, MA, USA)), Sheep Blood (Yuanye Bio-Technology in Shanghai, China), Haemophilus Test Medium Base (HTM, HalingBio in Shanghai, China), Tryptic Soy Broth (TSA, BD (Franklin Lakes, NJ, USA)), Cation Adjusted Muller-Hinton broth (CAMHB, BD), 7H9 broth (BD), OADC (BD), glycerol (Sigma), Tyloxapol (Sigma), and Chocolate Agar (BD) were from commercial sources as indicated. Rifampicin (RIF, Sigma), Ciprofloxacin (CIP, MedChemExpress (South Brunswick, NJ, USA)), Alamar Blue^TM^ Cell Viability Reagent from Thermo Fisher, and other chemical reagents were purchased.

### 3.2. Detection of AN11251 in Biological Samples

An aliquot of 40 µL AN11251 in DMSO solution was mixed with acetonitrile 200 µL (containing Labetalol, tolbutamide, Verapamil, dexamethasone, glyburide, and Celecoxib 100 ng/mL as internal standard for each) in a 96-well plate. Then the mixture was vortexed for 10 min at 800 rpm and centrifuged for 15 min at 3220× *g* at 4 °C. An aliquot of 50 µL supernatant was transferred to another clean 96-well plate and centrifuged for 5 min at 3220× *g* at 4 °C. The supernatant was directly injected for LC-MS/MS analysis. The instrument was LC-MS/MS-CS-Triple Quad 6500 plus. Chromatographic separations were carried out using ACQUITY UPLC HSS T3 LC column (1.8 μm × 2.1 × 50 mm). The mobile phases were 0.1% formic acid in water (A) and 0.1% formic acid in acetonitrile (B). The analytes were eluted using mobile phase B of 20% with a linear increase to 95% over 1 min and maintained for 0.4 min, followed by a return to the starting solution mixture in 0.1 min. The flow rate was 0.6 mL/min and the injection volume was 10 µL. The retention time of AN11251 was 0.99 min, and the retention time of celecoxib was 1.02 min. Negative ion electrospray tandem mass spectrometric analysis was carried out at unit resolution with collision-induced dissociation and selective reaction monitoring [12]. The MS analysis used SRM detection. The calculated *m*/*z* value of AN11251 as [M − H] was 527.2, and the found *m*/*z* value was 573.3 for [M − H + HCOOH]. The calculated *m*/*z* value of the internal standard celecoxide was 316.0, and the found *m*/*z* value for [M − H + HCOOH + H_2_O] was 380.0.

### 3.3. Plasma Protein Binding

Plasma protein binding of AN11251 was determined by equilibrium dialysis using an HT-Dialysis plate (Model HTD 96b) and the dialysis membrane (molecular weight cut off 12–14 KDa) in triplicate. Human plasma (BIOMEX GmbH) was mixed from more than 6 individuals and rat plasma was mixed from more than 10 rats. AN11251 was detected at the concentration of 2 µM, and Warfarin was the control compound. The samples were matched with opposite blank buffer to obtain a final volume of 100 µL with a volume ratio of the matrix: Dialysis Buffer (100 mM sodium phosphate and 150 mM NaCl, pH 7.4) (1:1) in each well. The stop solution (acetonitrile containing tolbutamide at 200 ng/mL, labetalol at 200 ng/mL) was added to the T0 sample of AN11251 and to the control sample. The plate was sealed and shaken at 800 rpm for 10 min. Then these T0 samples were stored at 4 °C pending further processing along with other post-dialysis samples. An aliquot of 100 µL loading matrix containing AN11251 or Warfarin was transferred to the donor side and 100 µL dialysis buffer was loaded to the receiving side of the well. The plate was rotated at 100 rpm in a humidified incubator with 5% CO_2_ at 37 °C for 4 h. At the end of the dialysis, all samples were further processed by protein precipitation for LC-MS/MS analysis. The %bound was calculated using the following equations:

%Unbound = 100 × F/T

%Bound = 100 − %Unbound

F = Free compound concentration as determined by the calculated concentration on the buffer side of the membrane.

T = Total compound concentration as determined by the calculated concentration on the matrix side of the membrane. 

### 3.4. Metabolic Stability in Microsomes

Microsomes from humans and rats were purchased from Corning (Shanghai, China) and Xenotech (Kansas City, KS, USA), respectively. The working solution of liver microsomes was prepared in 100 mM phosphate buffer at 0.56 mg/mL. The quench solution was cold (4 °C) acetonitrile (ACN) containing 200 ng/mL Verapamil and 200 ng/mL Imipramine as internal standards (IS). An amount of 445 µL of the microsomal working solution was transferred into the pre-warmed plates T120 (incubate time: 120 min) and NCF 120 (no co-factor NADPH regenerating system), then the plates were incubated at 37 °C for 10 min with constant shaking. An amount of 54 µL of the liver microsomes was transferred into a blank plate, and 6 µL NADPH and 180 µL quenching solution were added to the blank plate. The microsomal working solution (0.56 mg/mL) and the compound working solution (100 µM) were mixed 3 times thoroughly and 54 µL was immediately removed for the 0 min point. Then 44 µL NADPH cofactor was added to the incubation plate, and shaken at 37 °C for 120 min. At 15, 30, 60, 90, and 120 min, a 60 µL sample was transferred to the quenching solution and centrifuged at 4000 rpm for 20 min at 4 °C [6]. Then, 80 µL supernatant was transferred to the HPLC water and shaken for 10 min before LC-MS/MS analysis.

### 3.5. Metabolic Stability in Hepatocytes

Cryopreserved hepatocytes from male SD rats and humans were both purchased from Bioreclamation-IVT. The human cryopreserved hepatocytes were pooled from 10 human donors with a viability of 77.9% tested by trypan blue. The rats’ cryopreserved hepatocytes were pooled from 12 male SD (Sprague Dawley) rats, and the cell viability was 80.9%. The hepatocytes were diluted to 0.5 × 10^6^/mL cell suspension with pre-warmed incubation medium (ultra-pure water). For the T0 samples, the hepatocytes and AN11251 stock solution were mixed, and 25 µL of each sample was immediately transferred into a well containing 125 µL ice-cold stop solution. The hepatocytes were incubated with the AN11251 solution (20 µg/mL) in Williams’ Medium E at 37°C in a 95% humidified incubator at 5% CO_2_ to start the reactions with constant shaking at about 600 rpm. At 15, 30, 60, and 90 min, the samples were mixed and then 25 μL of each sample was transferred at each time point to a well containing 125 μL of ice-cold stop solution (acetonitrile containing 200 ng/mL tolbutamide and labetalol as internal standards) followed by mixing. The samples were vortexed on a plate shaker at 500 rpm for 10 min, and centrifuged at 3220× *g* for 20 min at 4 °C. The supernatants were transferred to ultra-pure water and analyzed by LC-MS/MS. The equation of first-order kinetics was used to calculate t_1/2_ and CL_int_:

C_t_ = C_0_ × e^−kt^

$$C_{t}=\frac{1}{2}C_{0},{\;t}_{1/2}=\frac{\ln2}{k}=\frac{0.693}{k}.$$

CL_int (hep)_ = k/million cells per mL

CL_int (liver)_ = CL_int (hep)_ × liver weight (g/kg body weight) × hepatocellularity

### 3.6. Pharmacokinetic Studies in Rats

The pharmacokinetic studies of AN11251 were performed in the formulation of PEG 400:PG:water = 55:25:20 as a clear solution. Animal husbandry procedures in this study were in compliance with the Animal Welfare Act, the National Research Council Guide for the Care and Use of Laboratory Animals (8th edition), and National Laboratory Animal Management Regulations (2017). The animals fasted overnight before administration. Each group had three male SD rats (Charles River Laboratories, bodyweight between 240 g and 250 g). For the intravenous (IV) group, a dose of 3 mg/kg of AN11251 was administered to the male rats by bolus infection. Blood samples were collected at the time points of 0.083 h, 0.25 h, 1 h, 2 h, 4 h, 8 h, 24 h, and 28 h. The oral (PO) group was dosed with AN11251 at 10 mg/kg in the same formulation as the IV group. Blood samples were collected at the time points of 0.25 h, 0.5 h, 1 h, 2 h, 4 h, 8 h, 24 h, and 28 h after dosing [13]. All the blood samples were centrifugated at 1000× *g* for 15 min and stored at a −80 °C refrigerator for LC-MS/MS analysis.

### 3.7. Pharmacokinetic Analysis

All the pharmacokinetic parameters were calculated from the drug plasma concentration–time data using Phoenix WinNonlin 6.3 and a non-compartmental model. The IV parameters included elimination half-time (T_1/2_), the volume of distribution steady state (Vdss), system clearance (Cl), mean residence time (MRT), the area under the plasma concentration–time curve from time 0 to infinity (AUC_0-inf_), and the AUC from time last extrapolated to infinity given as a percentage of AUC_0-inf_ (AUC_extra_). The PO parameters included the maximum concentration in plasma (C_max_), the time of maximum concentration in plasma (t_max_), and bioavailability (F). All the pharmacokinetic parameters were established using the mean plasma concentration data. If the adjusted rsq (linear regression coefficient of the concentration value on the terminal phase) was less than 0.9, T1/2 might not be accurately estimated. If the % AUC_Extra_ > 20%, AUC_0-inf_, Cl, MRT_0-inf_, and Vd_ss_ might not be accurately estimated. The oral bioavailability F in rats was determined using the following equation:

F = (AUC_0-inf, oral_/AUC_0-inf, iv_) × (Dose_iv_/Dose_oral_) × 100%

### 3.8. Bacterial Cell Culture

A total of twenty-four bacterial strains were chosen for testing in this study. The Gram-positive bacterial strains were *Staphylococcus aureus* ATCC 29213 (MSSA), *S. aureus* ATCC 700698 (MRSA), *S. aureus* SAU-0167 (MRSA, resistant to β-lactams, aminoglycosides, fluoroquinolones, and nitrofurantoin), *S. aureus* SAU-9922 (MRSA, resistant to β-lactams and fluoroquinolones), *Staphylococcus epidermidis* SEP-1024 (resistant to Aztreonam), *Streptococcus pneumoniae* SPN-1169 (resistant to Aztreonam, Cefoxitin, and Amikacin, etc.), *Streptococcus pyogenes* SPY-0253 (resistant to Aztreonam and Amikacin), *Enterococcus faecium* EFA-0221 (VRE, resistant to β-lactams, aminoglycosides, fluoroquinolones, and nitrofurantoin), *Enterococcus faecalis* EFA-9212 (resistant to some β-lactams including cephalosporins, aminoglycosides, and nitrofurantoin), and *E. faecalis* ATCC 29212. The strains with an ATCC code were purchased from American Type Culture Collection (ATCC) (Manassas, VA, USA) and the others were stored in HD Biosciences (Shanghai, China). Gram-negative strains were *Haemophilus influenzae* ATCC 49247, *Acinetobacter baumannii* ATCC BAA-1605, *Stenotrophomonas maltophilia* STM-0001 (resistant to β-lactams, aminoglycosides, and nitrofurantoin, from HD Bio), *Klebsiella pneumoniae* ATCC BAA-1705, *Pseudomonas aeruginosa* ATCC 27853, *Escherichia coli* ATCC 25922, and *Acinetobacter baumannii* ATCC 17978. Mycobacterial strains used were *Mycobacterium bovis* BCG Pasteur 1173P2, *Mycolicibacterium smegmatis mc*^2^155 ATCC 700084, *Mycobacterium tuberculosis* H37Rv ATCC 27294 (in Beijing Chest Hospital), *Mycobacterium abscessus*-GDI, and three clinical isolates: *M. abscessus* C16, C28, C58 (Beijing Children’s Hospital).

Antibacterial susceptibility tests were performed according to the Performance Standards for Antimicrobial Susceptibility Testing (M100, 29th Edition, 2019) published by the Clinical Laboratory Standards Institute (CLSI). The bacterial strains were first recovered from a −80 °C frozen stock on appropriate medium agar plates. Specifically, blood TSB agar plates were used for *Streptococcus* spp. and *Enterococcus* spp., chocolate agar plates for *H. influenzae*, and TSB agar plates for other strains. Plates were incubated at 37 °C with (*H. influenzae*, *Streptococcus* spp. and *Enterococcus* spp.) or without 5% CO_2_ for 24 h. Mycobacterial strains were recovered by thawing the frozen stock with fresh 7H9 broth medium supplemented with 10% OADC, 0.2% glycerol, and 0.05% tyloxapol and then shaking at 100 rpm at 37 °C.

### 3.9. Minimum Inhibitory Concentration (MIC) and Cytotoxicity Determination

Assay plates were prepared by dispensing stock solution of AN11251 or positive controls in a two-fold serial dilution starting from 32 µg/mL into 96-well cell plates. For anti-mycobacterial tests, the starting concentration of AN11251 was 20 µg/mL. The final volume in each well was made up to 2 µL with DMSO. CAMHB medium for Gram bacterial strains was prepared and sterilized. Inoculation suspension was made by resuspending fresh colonies in sterile saline (0.9% NaCl) and diluting to OD_600_ about 0.15. Then the suspension was further diluted 1:300 with each bacterial optimum medium to achieve ~2 × 10^5^ CFU/mL. Specifically, *H. influenzae* was with HTM broth, *Streptococcus* spp. and *Enterococcus* spp. with CAMHB supplemented with sheep blood, and CAMHB broth for other bacterial strains. An amount of 98 µL of each diluted bacterial inoculation was dispensed to the assay plates that had the compound. Medium containing 2% DMSO served as a negative control, while media containing Rifampicin or Ciprofloxacin served as positive controls. The plates were incubated for 20 h at 37 °C. The lowest concentration of the essential inhibited any increases visible to the naked eye, which were noted as MIC. Determination of antimycobacterial MIC with Microplate Alamar Blue Assay (MABA) followed the Mycobacteria Protocols (2015, 3rd Ed. T Parish). Recovered mycobacterial strains were transferred to fresh 7H9 broth medium and grown to the log phase OD_600_ ~0.6–0.8. The mycobacterial culture was further diluted with 7H9 broth medium until OD_600_ 0.01. An amount of 200 µL diluted inoculation was dispensed into each well of the 96-well assay plates. The 7H9 medium with 1% DMSO served as a negative control and Rifampicin and Isoniazid as the positive controls. Assay plates with *M. smegmatis* and *M. abscessus* were incubated at 37 °C for 72 h, while *M. bovis* BCG and *M. tuberculosis* H37Rv were incubated for 7 days. Afterward, 50 µL Alamar Blue reagent prepared with the commercial stock and 10% Tween 80 in a volume ratio of 1:1 was added to each well, followed by 20 h of incubation at 37 °C. Fluorescence at excitation 544 nm and emission 590 nm was measured. The cytotoxicity of AN11251 was assayed against the Vero6 cell line by a 10-point series with 2-fold dilution of the compound in the range of 40 to 0.078 µM in triplicate in a 96-well microplate. The luminescence generated with CellTiter-Glo kit (Promega) was recorded after 48 h incubation and the cytotoxic concentration (CC50) was determined. Data were analyzed with GraphPad Prism 9.5.

### 3.10. Prediction of the PK Parameters in Humans

The human systemic clearance and volume of distribution were predicted through allometric scaling reported by Johnson & Johnson [14]. Allometric scaling is empirically based on the similarity of anatomy and physiology in mammals. The body weight, brain weight, and maximum lifespan of different animals all influence the animal’s distribution and elimination. The rat is the preferred species for evaluating the PK profile of drug candidates in early stages. The systemic clearance and the volume of distribution of humans have a strong relationship with those of rats. Based on the investigation of a large and diverse set of drugs, a fixed exponent allometric scaling method could be used to predict human in vivo PK parameters.

We used two approaches to predict the human PK parameters from rat in vivo PK data. Both strategies were consistent to the standard allometric equation:

Y = a W^b^

where Y is the human PK parameter such as systemic clearance and volume of distribution. W is the body weight, a is the allometric coefficient, and b is the allometric exponent.

The first method uses the log–log regression technique to express the relationship between human and rat PK parameters as follows:

LogY_human_ = α (LogY_rat_) + β

where Y is the PK parameter, such as systemic clearance and volume of distribution, α is the slope, and β is the intercept.

The second method assumes the allometric coefficient and exponent are fixed values, and the PK values scaled from rats to human can be expressed as follows:

Y_human_ = Y_rat_ (W_human_/W_rat_)^b^

where Y is the PK parameter systemic clearance and volume of distribution. W_human_, the human body weight, is assumed to be 70 kg. W_rat_, the rat body weight, is assumed to be 0.25 kg in the calculations. The b allometric exponents were between 0–1 after calculation.

### 3.11. Systemic Clearance

Using the first method, the unit of the experimental clearance is mL/min/kg. There is a reasonable relationship between human and rat systemic clearance values:

Log CL_human_ = 0.8653 (LogCL_rat_) − 0.5659

Using the second method, the exponent is defined as 0.67, a human body weight is 70 kg, and a rat body weight is 0.25 kg. The equation is derived as follows:

CL_human_ = CL_rat_ (W_human_/W_rat_)^b^ = 43.61 CL_rat_ (L/hr)

This equation can be expressed as follows after calculation:

CL_human_ = 0.1588 CL_rat_ (L/hr/kg)

Based on the analysis results, the two methods are equivalent. Thus, we used the following simplified allometric scaling equation to predict human systemic clearance:

CL_human_ = 40 CL_rat_ (L/hr)

### 3.12. Volume of Distribution

Using the first method, the unit of volume of distribution was L/kg. There was a good correlation between human volume of distribution and rat:

Log Vss_human_ = 0.8270 (Log Vss_rat_) − 0.1797

Using the second method, the exponent was fixed to be 0.93, the human body weight was 70 kg, and the rat body weight was 0.25 kg. The equation could be derived as follows:

Vss_human_ = Vss_rat_ (W_human_/W_rat_) ^b^ = 188.74 Vss_rat_ (L)

The equation could be expressed as follows after calculation:

Vss_human_ = 0.6741 Vss_rat_ (L/kg)

Based on the calculation results, the two prediction methods are equivalent. Thus, we used the simplified allometric scaling equation to predict human volume of distribution:

Vss_human_ = 200 Vss_rat_ (L)

### 3.13. Prediction the Human Dose Using PK/PD Modeling

The human dose for efficacy was predicted by one-compartment PK/PD modeling [15]. The dosing frequency was twice a day (BID) and lasted for four days. The PK/PD modeling predicts the dose using the equation as follows:

C = ka × F × X_0_ × (e^−kel×t^ − e^−ka×t^)/(V × (ka − k))

A = ka × F × X_0_/(V × (ka − k))

Ka: absorption rate constant

F: bioavailability

X_0_: dose

kel: elimination rate constant

V: apparent distribution volume

At the first day 12 h, the equation is as follows:

C = A × (e^−kel×t^ − e^−ka×t^)

12–24 h:

C = A × (e^−kel×(t−12)^ − e^−ka×(t−12)^) + A × (e^−kel×t^ − e^−ka×t^)

## 4. Conclusions

In conclusion, this work provides a further study on the potency and PK profile of AN11251. Because of the incorporated benzoxaborole at the C14 of pleuromutilin, AN11251 exhibits a good balance on the ADME properties and antibacterial activities [11]. To our delight, AN11251 has a moderate human hepatic (64.8 µL/min/10^6^) and high plasma protein binding (PPB: 0.976), which would generate a good systemic clearance. The high lipophilicity (log P = 4.5) is beneficial to the good permeability (Papp = 14.1 × 10^−6^ cm/s) and bioavailability. The high plasma protein binding helps in reducing in vivo metabolism. When AN11251 was administrated to rats by the intravenous route (3 mg/kg), we observed moderate to good clearance (CL = 19.8 mL/min/kg) and good exposure (AUC_0–24_ = 2550 ng. h/mL). Its primary metabolite results from the hydroxylation at C (2) site. There have been plenty of modifications for improving the PK properties of pleuromutilin. Substituting C14 with leucine and the incorporation of the carbonyl group at C3 were shown to increase the half-life, but the AUC was very low in the mice PK profile, which made that compound unlikely to be a good anti-TB drug candidate [23]. Modification of pleuromutilin with pyridine-thiazole at the C14 site could generate a compound with promising anti-MRSA isolate activity, but may result in a poor PK profile as well, such as high systemic clearance and low exposure. Heteroaromatic substitution in the C14 side chain of pleuromutilin presents different in vitro and in vivo properties, indicating that minor structure differences in the substituents on the C14 would have considerable influence on the PK profile [24].

AN11251 has been described as an anti-*Wolbachia* drug candidate [13], with a low MIC from 0.79 µg/mL to 7.9 µg/mL. Through PK/PD modeling, the human dose is predicted to be only 1.7 mg twice a day, or 14.7 mg once a day based on the EC50 of the *Wolbachia*-infected LWD1 cells. The SAR study demonstrated that the optimal activity was obtained by linking benzoxaborole to the pleuromutilin core, which impacts the potency and PK properties. Secondly, leucine modification of pleuromutilin was shown to be effective against replicating and non-replicating TB bacteria [23]. Here, AN11251 shows anti-TB activity with MIC at 0.952 µg/mL, leading to the human dose predicted to be 2012 mg twice a day. Furthermore, pyridine-thiazole-pleuromutilin has been evaluated against MRSA with MIC from 4 µg/mL to 64 µg/mL [25]. The anti-Gram-positive activity of AN11251 was also investigated and shown to be good to excellent. The MIC value ranges from less than 0.039 µg/mL to 0.5 µg/mL against a broad spectrum of G+ bacterial species, and the effective human dose could thus be from 84 mg to 2012 mg twice a day. All the results demonstrated that AN11251 has a good PK profile and has the potential to be developed as a preclinical anti-*Wolbachia* and anti-Gram-positive candidate.

## Figures and Tables

**Table 1:** Pharmacokinetic profile of AN11251.

Species	Route	Dose (mg/kg)	CL (mL/min/kg)	Vdss (L/kg)	AUClast (ng. h/mL)	Cmax (µM)	Tmax (h)	MRT (h)	T1/2 (h)	F (%)
Rat	IV	3	19.8	1.44	2503	4089	0	1.04	1.75	19.2
	PO	10			1453	819	0.25	2.26	2.39	
mouse	IV	5	8.4	4.186	8850	3210	0		9.1	61
	PO	10			12,100	1600	0.083		8.41	

**Table 2:** The MIC of AN11251 and antibiotic drugs against bacterial pathogens. (--, not measured).

Groups	Strains	AN11251 (µg/mL)	Rifampicin (µg/mL)	Ciproflox (µg/mL)	Amikacin (µg/mL)
Gram- positive	*S. aureus* ATCC 29213 (MSSA)	<0.031	0.0063	--	8
	*S. aureus* ATCC 700698 (MRSA)	<0.031	≤0.031	--	>32
	*S. aureus* SAU-0167 (MRSA)	0.063	≤0.031	>32	>32
	*S. aureus* SAU-9922 (MRSA)	0.063	≤0.031	>32	2
	*S. epidermidis* SEP-1024	0.063	≤0.031	0.25	≤1
	*S. pneumoniae* SPN-1169	0.5	≤0.031	1	>32
	*S. pyogenes* SPY-0253	0.063	0.063	0.25	32
	*E. faecium* EFA-0221 (VRE)	0.125	4	>32	>32
	*E. faecalis* EFA-1299	4	1	0.25	>32
	*E. faecalis* ATCC 29212	5	2	--	>32
Gram- negative	*H. influenzae* ATCC 49247	2	0.25	≤0.031	--
	*A. baumannii* ATCC 17978	20	2	--	2
	*A. baumannii* ATCC BAA-1605	32	4	>32	--
	*K. pneumoniae* ATCC BAA-1705	>32	>32	--	>32
	*P. aeruginosa* ATCC 27853	>32	>32	--	8
	*E. coli* ATCC 25922	>32	8	--	8
	*S. maltophilia* STM-0001	>32	16	2	>32
Myco- bacteria	*M. tuberculosis H37Rv* ATCC27294	0.952	0.031	--	--
	*M. bovis BCG* (Pasteur 1173P2)	2.5	≤0.031	--	--
	*M. smegmatis* mc^2^155	>20	13.26	--	--
	*M. abscessus*-GDI	>20	>20	--	6.38
	*M. abscessus*-C16	>20	>20	--	12.71
	*M. abscessus*-C28	>20	>20	--	14.46
	*M. abscessus*-C58	>20	>20	--	12.82

## Data Availability

Not applicable.

## References

[1] Kavanagh F., Hervey A., Robbins W.J. (1951). Antibiotic Substances from Basidiomycetes: VIII. Pleurotus Multilus (Fr.) Sacc. and Pleurotus Passeckerianus Pilat. Proc. Natl. Acad. Sci. USA.

[2] Paukner S., Riedl R. (2016). Pleuromutilins: Potent Drugs for Resistant Bugs—Mode of Action and Resistance. Cold Spring Harb. Perspect. Med..

[3] Heidtmann C.V., Voukia F., Hansen L.N., Sørensen S.H., Urlund B., Nielsen S., Pedersen M., Kelawi N., Andersen B.N., Pedersen M. (2020). Discovery of a Potent Adenine–Benzyltriazolo–Pleuromutilin Conjugate with Pronounced Antibacterial Activity against MRSA. J. Med. Chem..

[4] Ling C., Fu L., Gao S., Chu W., Wang H., Huang Y., Chen X., Yang Y. (2014). Design, synthesis, and structure-activity relationship studies of novel thioether pleuromutilin derivatives as potent antibacterial agents. J. Med. Chem..

[5] Garmyn A., Vereecken M., Degussem K., Depondt W., Haesebrouck F., Martel A. (2017). Efficacy of tiamulin alone or in combination with chlortetracycline against experimental Mycoplasma gallisepticum infection in chickens. Poult. Sci..

[6] Siricilla S., Mitachi K., Yang J., Eslamimehr S., Lemieux M.R., Meibohm B., Ji Y., Kurosu M. (2017). A New Combination of a Pleuromutilin Derivative and Doxycycline for Treatment of Multidrug-Resistant *Acinetobacter baumannii*. J. Med. Chem..

[7] Drews J., Georgopoulos A., Laber G., Schutze E., Unger J. (1975). Antimicrobial activities of 81.723 hfu, a new pleuromutilin derivative. Antimicrob. Agents Chemother..

[8] Laber G., Schütze E. (1975). In Vivo Efficacy of 81.723 hfu, a New Pleuromutilin Derivative against Experimentally Induced Airsacculitis in Chicks and Turkey Poults. Antimicrob. Agents Chemother..

[9] Goldstein E.J., Citron D.M., Merriam C.V., Warren Y.A., Tyrrell K.L., Fernandez H.T. (2006). Comparative in vitro activities of retapamulin (SB-275833) against 141 clinical isolates of Propionibacterium spp., including 117 P. acnes isolates. Antimicrob. Agents Chemother..

[10] Jones R.N., Fritsche T.R., Sader H.S., Ross J.E. (2006). Activity of Retapamulin (SB-275833), a Novel Pleuromutilin, against Selected Resistant Gram-Positive Cocci. Antimicrob. Agents Chemother..

[11] Jacobsson S., Paukner S., Golparian D., Jensen J.S., Unemo M. (2017). In Vitro Activity of the Novel Pleuromutilin Lefamulin (BC-3781) and Effect of Efflux Pump Inactivation on Multidrug-Resistant and Extensively Drug-Resistant Neisseria gonorrhoeae. Antimicrob. Agents Chemother..

[12] Jacobs R.T., Lunde C.S., Freund Y.R., Hernandez V., Li X., Xia Y., Carter D.S., Berry P.W., Halladay J., Rock F. (2019). Boron-Pleuromutilins as Anti-*Wolbachia* Agents with Potential for Treatment of Onchocerciasis and Lymphatic Filariasis. J. Med. Chem..

[13] Ehrens A., Lunde C.S., Jacobs R.T., Struever D., Koschel M., Frohberger S.J., Lenz F., Fendler M., Turner J., Ward S. (2020). In vivo efficacy of the boron-pleuromutilin AN11251 against Wolbachia of the rodent filarial nematode Litomosoides sigmodontis. PLOS Neglected Trop. Dis..

[14] Caldwell G.W., Masucci J.A., Yan Z., Hageman W. (2004). Allometric scaling of pharmacokinetic parameters in drug discovery: Can human CL, Vss and t1/2 be predicted fromin-vivo rat data?. Eur. J. Drug Metab. Pharmacokinet..

[15] Derendorf H., Lesko L.J., Chaikin P., Colburn W.A., Lee P., Miller R., Powell R., Rhodes G., Stanski D., Venitz J. (2000). Pharmacokinetic/Pharmacodynamic Modeling in Drug Research and Development. J. Clin. Pharmacol..

[16] Smith D.A., Di L., Kerns E.H. (2010). The effect of plasma protein binding on in vivo efficacy: Misconceptions in drug discovery. Nat. Rev. Drug Discov..

[17] Di L., Keefer C., Scott D., Strelevitz T.J., Chang G., Bi Y.-A., Lai Y., Duckworth J., Fenner K., Troutman M.D. (2012). Mechanistic insights from comparing intrinsic clearance values between human liver microsomes and hepatocytes to guide drug design. Eur. J. Med. Chem..

[18] Smith D.A., Beaumont K., Maurer T.S., Di L. (2018). Clearance in Drug Design. J. Med. Chem..

[19] Tess D.A., Ryu S., Di L. (2022). In Vitro-in Vivo Extrapolation of Hepatic Clearance in Preclinical Species. Pharm. Res..

[20] Novak R. (2011). Are pleuromutilin antibiotics finally fit for human use?. Ann. N. Y. Acad. Sci..

[21] Novak R., Shlaes D.M. (2010). The pleuromutilin antibiotics: A new class for human use. Curr. Opin. Investig. Drugs.

[22] Li X., Lund C.S., Jacobs R.T., Hernandez V.S. (2017). Boron-Containing Small Molecules Cross-Reference to Related Applications.

[23] Lemieux M.R., Siricilla S., Mitachi K., Eslamimehr S., Wang Y., Yang D., Pressly J.D., Kong Y., Park F., Franzblau S. (2018). An antimycobacterial pleuromutilin analogue effective against dormant bacilli. Bioorg. Med. Chem..

[24] Panchal R.G., Ulrich R.L., Lane D., Butler M.M., Houseweart C., Opperman T., Williams J.D., Peet N.P., Moir D.T., Nguyen T. (2009). Novel Broad-Spectrum Bis-(Imidazolinylindole) Derivatives with Potent Antibacterial Activities against Antibiotic-Resistant Strains. Antimicrob. Agents Chemother..

[25] Xia J., Xin L., Li J., Tian L., Wu K., Zhang S., Yan W., Li H., Zhao Q., Liang C. (2023). Discovery of Quaternized Pyridine-Thiazole-Pleuromutilin Derivatives with Broad-Spectrum Antibacterial and Potent Anti-MRSA Activity. J. Med. Chem..

